# Extracorporeal photopheresis therapy for graft-versus-host disease with atypical neurological manifestations: a case report

**DOI:** 10.1007/s00277-026-06971-9

**Published:** 2026-03-28

**Authors:** Linda Anelli, Daniele Avenoso

**Affiliations:** Unit of Blood Diseases and Bone Marrow Transplantation, Department of Clinical and Experimental Science, University of Brescia, ASST Spedali Civili di Brescia, Piazzale Spedali Civili 1, Brescia, 25123 Italy

**Keywords:** ECP, GVHD, allo-HSCT, CMV, fungal infection

## Abstract

Uncommon manifestations of graft-versus-host disease are a clinical challenge due to the absence of clear diagnostic criteria and treatment strategies. Herein, we present the case of a patient with neurological manifestations of GVHD that successfully resolved after extracorporeal photopheresis.

## Introduction

Graft-versus-host disease (GVHD) is the main reason for morbidity and mortality after allogeneic haematopoietic stem cell transplant (allo-HSCT) [1]. Recently the EBMT-ELN updated its guidelines for the prophylaxis and treatment of GVHD [2]. If on one had there is a clear stratification of GVHD severity and a relatively straightforward treatment algorythm, there is still lack of certain diagnostic criteria and treatment options for uncommon GVHD manifestations, such as central nervous system (CNS) inflammation [3, 4]. In case of failure of systemic steroids, Extracorporeal Photopheresis (ECP) is an effective and recommended second line treatment [5] as well as ruxolitinib [2], for both acute and chonic GVHD.

Following steroid failure, clinicians offer ECP since 1994 occasionally associated to other immune suppressive agents [6]. Only in the last decade, transplant physician can offer either target therapy or monoclonal antibodies for the treatment of steroid-refractory GVHD (SR-GVHD) [7–9].

Within the SR-GVHD, at least four different groups of patients can be identified: (1) those with GVHD progression after three days of systemic therapy; (2) those with lack of response following five days of treatment; (3) progression to a new organ; (4) recurrence of GVHD during or after steroid tapering. This clinical scenario is even more challenging when allo-HSCT patients develop uncommon GVHD manifestations with a background of opportunistic infections (both viral and fungal); indeed a successful SR-GVHD treatment is a deliquate balance between enough immune suppression to suppress organ inflammation without hampering immune system surveillance towards both opportunistic infections and the haematological malignancy that was the indication for allo-HSCT. In this particular scenario, ECP is likely the best therapeutic option due to its intrinsic immune-modulatory properties [10, 11].

## Case presentation

Herein we present the case of a 60-years-old man diagnosed with myelodysplastic syndrome (MDS) with SF3B1 mutation, who underwent reduced intensity allo-HSCT from a matched unrelated donor. GVHD prophylaxis consisted of Alemtuzumab 60 mg and Ciclosporin 3 mg/kg. On day + 29 the patient developed bilateral facial nerve paralysis. Neurological examination revealed symmetrical peripheral facial palsy involving both upper and lower facial muscles, with impaired forehead elevation, incomplete eyelid closure, and bilateral flattening of the nasolabial folds. Cerebrospinal fluid investigations were negative for leukaemic localization and infectious etiologies. Brain magnetic resonance imaging (MRI) was performed and showed no structural abnormalities, demyelinating lesions, ischemic events, or contrast-enhancing findings involving the facial nerve pathways. Acute skin GVHD grade II developed on day + 30, and systemic prednisolone 1 mg/kg/day was initiated, leading to complete resolution of cutaneous manifestations. Steroids were subsequently tapered after achievement of complete response; bilateral facial nerve palsy persisted without clinical improvement. Despite letermovir prophylaxis the patient experienced three episodes of CMV reactivation, which were managed with multiple cycles of valganciclovir. Concurrently, there was progression of a known fungal nodule, diagnosed pre-HSCT. Despite the dermatological resolution of aGVHD, the patient still presented bilateral facial palsy. On day + 240 the patient presented with clinical features diagnostic for chronic GVHD. Cutaneous involvement was characterized by fibrotic skin changes consistent with sclerotic GVHD, associated with lichenoid lesions. Oral examination revealed lichen-type mucosal involvement. Musculoskeletal manifestations included joint stiffness and reduced range of motion suggestive of fascial/articular GVHD. Liver involvement was documented by altered liver function tests consistent with hepatic GVHD. Neurological deficits remained stable over time and were still present at the time. According to the 2014 NIH consensus criteria, chronic GVHD was classified as moderate in global severity. Organ-specific involvement included sclerotic and lichenoid skin manifestations (skin score 2), oral lichen-type mucosal involvement (score 1–2), fascial/joint restriction with reduced range of motion (score 2), and hepatic involvement documented by liver function test abnormalities (score 1). Due to his severe impairment in quality of life and his known infectious complications (previous CMV reactivations and pulmonary fungal infection), a short course of steroids was given as bridge to ECP with Cellex Therakos™ planned to be given three times a week for four weeks and then tapered with two weekly sessions every other week.

The patient showed a progressive improvement in performance status after the initiation of steroid therapy, without changes within the GVHD manifestations. Subsequently, with ongoing ECP treatment, GVHD manifestations steadily improved over time. Oral mucosal involvement, skin inflammation, and liver function abnormalities progressively resolved. In parallel, musculoskeletal manifestations also improved, with reduction of joint stiffness and progressive recovery in range of motion. Neurological involvement followed a similar trajectory, with gradual resolution of facial nerve palsy. At six weeks after the start of ECP, oral mucosa involvement, body weight, and skin inflammation normalized. Three weeks later, skin involvement and liver function tests showed further improvement, with complete resolution of the left-sided facial palsy, while mild right-sided facial weakness persisted but continued to improve progressively during ongoing extracorporeal photopheresis therapy.

At 14 weeks after ECP initiation, only minimal residual left facial palsy persisted. ECP therapy was therefore continued and gradually tapered until discontinuation.

## Discussion

This case shows the importance of the treatment with ECP to manage long-term consequences of SR-GVHD in a patient with important infective comorbidities; it also offers a possible point of speculation: the bilateral facial palsy was indeed an acute GVHD manifestations that was steroid refractory. A major limitation of this report is the inability to establish a definite causal relationship between GVHD and the observed bilateral facial nerve palsy. Idiopathic facial palsy (Bell’s palsy) represents the most common etiology in the general population and is frequently self-limiting, with spontaneous recovery occurring within weeks to months. Therefore, a coincidental occurrence cannot be formally excluded. Nevertheless, the temporal proximity to allo-HSCT, the absence of infectious or malignant involvement on cerebrospinal fluid analyses, the concomitant development of multi-organ GVHD manifestations, and the progressive neurological improvement observed during extracorporeal photopheresis may collectively support an immune-mediated mechanism potentially related to GVHD. This observation should thus be interpreted as hypothesis-generating rather than confirmatory. It must be ackowledged that SR-GVHD remains a major unmet clinical need after HSCT. However, its current clinical definition encompasses a biologically and clinically heterogeneous spectrum of conditions [12]. Differences between moderate and severe cGVHD, as well as among steroid-refractory, steroid-dependent, and late steroid-responsive GVHD, likely reflect distinct immunobiological trajectories with variable sensitivity to second-line therapies. Current guidelines, which are largely based on GVHD grade and organ involvement, do not adequately address rare or atypical manifestations, including CNS involvement, which are underrepresented in clinical trials and lack evidence-based therapeutic recommendations. Within this framework, central nervous system involvement may be conceptualized among the spectrum of “atypical chronic GVHD” manifestations, as proposed by the 2020 NIH cGVHD Consensus Project [13], which recognized inflammatory and immune-mediated complications affecting non-classical target organs, including the nervous system. Although the precise mechanisms underlying neurological GVHD remain poorly defined, the observed response to ECP may be biologically plausible. Indeed, ECP is known to exert immunomodulatory rather than broadly immunosuppressive effects, promoting regulatory T-cell expansion, inducing tolerogenic dendritic cell phenotypes, and reducing alloreactive T-cell activity. These mechanisms may attenuate immune-mediated inflammatory damage within peripheral or central nervous system structures. Furthermore, the steroid-sparing profile of ECP may be particularly advantageous in patients with infectious vulnerability, allowing control of pathogenic immune responses without exacerbating opportunistic risk. In this setting, rigid algorithm-driven treatment strategies appear insufficient and should evolve toward more individualized approaches that incorporate disease kinetics, patterns of organ involvement, infectious comorbidities, and patient frailty. The present case suggests that early integration of ECP may be an effective therapeutic option even in unconventional GVHD presentations, allowing disease control while minimizing prolonged corticosteroid exposure in patients at high infectious risk.

## Conclusions

To our knowledge, this represents the first reported case of GVHD with CNS involvement successfully treated with ECP. Overall, this observation supports also the concept that the efficacy of ECP is not solely determined by the formal classification of steroid-refractory GVHD, but rather by timing of intervention and appropriate patient selection. Prospective studies and a critical reappraisal of current GVHD classifications are warranted to better capture disease heterogeneity and optimize therapeutic strategies and long-term outcomes.

## Data Availability

No datasets were generated or analysed during the current study.
